# Early Initiation of Thyroid Hormone Therapy Versus Watchful Waiting or Delayed Treatment or no Treatment in Subclinical Hypothyroidism: A Systematic Review and Meta-analysis Protocol

**DOI:** 10.12688/f1000research.179360.3

**Published:** 2026-09-29

**Authors:** Aravind P Gandhi, Kalyani P Deshmukh, Anupriya Kaliyappan, Akash Bang, Neha Gangane

**Affiliations:** 1 Department of Community Medicine, All India Institute of Medical Sciences - Nagpur, Nagpur, Maharashtra, India; 2 Technical Resource Centre, Centre for evidence-based guidelines, All India Institute of Medical Sciences - Nagpur, Nagpur, Maharashtra, India; 3 Department of Endocrinology & Metabolism, All India Institute of Medical Sciences - Nagpur, Nagpur, Maharashtra, India; 4 Department of Pediatrics, All India Institute of Medical Sciences - Nagpur, Nagpur, Maharashtra, India; 5 Department of Obstetrics and Gynecology, All India Institute of Medical Sciences - Nagpur, Nagpur, Maharashtra, India

**Keywords:** subclinical hypothyroidism, levothyroxine, pregnancy, adults, meta-analysis, evidence synthesis

## Abstract

**Background:**

The prevalence of subclinical hypothyroidism (SCH) in the general population is notably high, ranging from 4% to 20%, and varies according to sex and age. SCH has been reported to be associated with multiple adverse pregnancy outcomes in pregnant women. While the therapeutic decision for overt hypothyroidism is fairly straightforward, treatment of SCH, especially the timing of the initiation of therapy, has been a point of discussion.

**Objective:**

To assess the effect of early initiation of treatment (thyroid hormone therapy) compared to watchful waiting, delayed initiation, or no treatment on progression to overt hypothyroidism, prevention of complications, and associated adverse outcomes through a “systematic review and meta-analysis (SRMA)”.

**Methods:**

The SRMA protocol adhered to the “Preferred Reporting Items for Systematic reviews and Meta-Analyses Protocols (PRISMA-P 2015)” guidelines. Only “randomized controlled trials (RCTs)” will be included. The databases “PubMed, Scopus, EMBASE, and the Cochrane Library” will be searched from inception until 30.12.2025. Two stages (title abstract followed by full-text review) and two-pass screening (two authors independently) with a third reviewer adjudication of conflicts will be adopted. Data will be extracted, and the risk of bias will be assessed independently by two authors using a process to resolve differences. The risk of bias for RCTs will be assessed using Cochrane ROB 2.0. Pooled estimates will be calculated for meta-analysis. Subgroup analysis and meta-regression will be performed if heterogeneity was present. The certainty of the evidence will be ascertained through “GRADE (Grading of Recommendations, Assessment, Development, and Evaluation)”.

**Intended outcomes:**

The SRMA can inform the framing of clinical guidelines for SCH management for all population groups and also intends to bring out gaps in the existing literature for future studies.

PROSPERO ID: CRD420251270021 (Date: 08 February 2026)

## 1. Introduction

Hypothyroidism is a major problem which affects 0.3 to 12% of the global population ^1^ and contributes significantly to morbidity. ^2^ Broadly, it includes overt, central, and subclinical hypothyroidism, depending on the varied combinations of thyroid function test (TFT) markers. “Subclinical hypothyroidism (SCH)” is identified when “serum-free thyroxine (FT4)” and “free triiodothyronine (FT3)” levels are normal, but “thyroid-stimulating hormone (TSH)” levels are elevated. ^3^ The characteristic laboratory finding of subclinical hypothyroidism is an increased TSH level along with a normal T4 level. ^4^ While it is typically regarded as an asymptomatic condition, some patients may exhibit non-specific symptoms that could indicate hypothyroidism. The prevalence of SCH in the general population is notably high (4% to 20%) and varies by sex and age. It is more common in individuals over 60 years of age, with a prevalence of approximately 15% in women and 8% in men. ^5^ Of individuals with grade 1 subclinical hypothyroidism, 60% experienced a return of thyrotropin to normal levels within a five-year period. The yearly likelihood of advancing to overt hypothyroidism in these patients ranges from 2% to 4%, and is influenced by the presence of TPO antibodies. Among older adults (aged 65 and above), 46% of those with “grade 1 subclinical hypothyroidism” (TSH levels ranging from 4.5 to 6.9 mU/L) saw their thyrotropin normalize within two years, in contrast to only 7% of those with grade 2 subclinical hypothyroidism. ^6^ SCH in pregnancy significantly progresses to overt hypothyroidism in the post-pregnancy period (within 5 years). ^7^ In pregnant women, SCH has been reported to be associated with multiple “adverse pregnancy outcomes (APOs),” such as preeclampsia and preterm birth, among others. ^8, 9^ Higher risk for pregnancy loss, hypertensive disorders of pregnancy and placental abruption in SCH group has also been reported in the past meta-analyses. ^10, 11^

While the therapeutic decision for overt hypothyroidism is fairly straightforward, treatment of SCH, especially the timing of the initiation of therapy, has been a point of discussion. In pregnant women, levothyroxine (LT4) treatment has been shown to reduce APOs, such as preterm birth, gestational hypertension, pregnancy loss, and neonatal mortality. ^12– 14^ Effect of LT4 therapy in specific sub-groups such as recurrent pregnancy loss is a gap to be explored in evidence synthesis. ^15^ Although multiple meta-analyses have reported the efficacy of LT4 therapy on pregnancy outcomes, ^16^ relatively long-term outcomes such as neurodevelopment in infancy have not been reported. For adult SCH patients, levothyroxine treatment has been recommended since low-quality evidence was present for the critical outcomes (as of 2019). ^17^ Peng In their systematic review of studies conducted until April 2020, Peng et al. reported a differential pattern of LT4 association with mortality among older and younger adults. ^18^ Treatment for SCH also needs to factor in the TPOAb positive and levels of TSH at diagnosis, since these factors have been shown to increase the risk of progression of SCH to overt hypothyroidism. ^9, 19^ An updated pooled systematic review on the effect of early initiation of thyroid hormone therapy among the adults (non-pregnant women, young adults and old adults) is not available. In addition, important effect modifiers such as age, sex, comorbidity, initial TSH levels of patients (as reported by the studies), symptom status, and the type of control group used (placebo/no treatment/watchful waiting/delayed initiation) need to be explored to ascertain any differential effects of LT4 therapy among patients with SCH.

Considering the current burden of SCH surpassing overt hypothyroidism, and that this hidden thyroid dysfunction can lead to complications, reduced quality of life, productivity, and lack of a comprehensive systematic review assessing the impact of early initiation of thyroid hormone therapy in SCH, including all population groups, the current “systematic review and meta-analysis (SRMA)” is being proposed. The objective of this systematic review is to assess the effect of early initiation of thyroid hormone therapy on the prevention of complications, adverse events, and quality of life among SCH patients.

## 2. Protocol

The SRMA protocol adhered to the “Preferred Reporting Items for Systematic reviews and Meta-Analyses Protocols (PRISMA-P 2015)” guidelines (Extended Data: Table S1). The review is registered in the PROSPERO database (CRD420251270021).

### 2.1 Research question

In individuals with subclinical hypothyroidism (P), what are the comparative effects of early initiation of treatment (thyroid hormone therapy) (I) compared to watchful waiting, delayed initiation, or no treatment (C) on progression to overt hypothyroidism and prevention of complications and their associated adverse outcomes (O)? The detailed eligibility criteria for the review are listed in Table 1.

**Table 1.** Eligibility criteria for the studies.

PICO	Eligibility criteria
Population (P)	Individuals diagnosed with SCH (defined as elevated TSH above the laboratory reference range with normal free T4) Subgroups: • By TSH bands/levels: 4 to 7 mIU/L, 7 to 10 mIU/L, >10 mIU/L, or any other cut-off specified by the included studies for the diagnosis of subclinical hypothyroidism • By age bands: Adults (<60 years vs. 60 years) • By symptom Status: Symptomatic vs asymptomatic individuals • By life stage and reproductive status • Neonates (pre-term v/s term) • Children (family history, obesity and neurodevelopmental delay or any other) • Pregnant women (trimester-specific), periconceptional period, post-partum (lactating mother), females planning pregnancy, women of reproductive age (WRA) • By comorbid conditions: Cardiovascular disease, infertility, psychiatric disorders, NCDs, anaemia, obesity, metabolic syndrome • By autoimmune status: Individuals with positive thyroid autoantibodies • By environmental or geographic context: Geographic or iodine status differences • By clinical features: Presence or absence of goitre
Intervention (I)	Initiation of LT4 therapy at time of SCH diagnosis (with or without any other treatment)
Comparator (C)	1. Watchful waiting/active surveillance (observation with periodic monitoring of TSH and clinical status) 2. Delayed initiation: Treatment initiated only after prespecified threshold are reached or symptoms developed 3. No treatment: if distinct from watchful waiting in study design
Outcomes (O)	Critical 1. Progression to overt hypothyroidism 2. Prevention of complications (e.g., maternal, fetal, neonatal, others, depending upon the subgroups) 3. Adverse effects of treatment 4. Mortality (all-cause and, where reported, cause-specific mortality) Important 1. Improvement in symptoms 2. Improvement in Health-related QoL 3. Health system outcomes (cost-effectiveness, feasibility, acceptability, equity)

Given the expected variability in diagnostic criteria for subclinical hypothyroidism across different populations, geographical settings, time periods, and evolving clinical guidelines, we will include all eligible studies, regardless of the diagnostic thresholds used, provided the study authors classify participants as having subclinical hypothyroidism.

Early initiation of treatment refers to thyroid hormone therapy initiated immediately following the diagnosis of subclinical hypothyroidism, without a planned period of observation or delayed treatment. Studies comparing this strategy with watchful waiting, delayed therapy initiation, or no treatment will be considered eligible. Where the timing of treatment initiation is not explicitly reported, classification will be based on the intervention description and treatment protocol. If the timing remains unclear after review of the full text and supplementary materials, the corresponding study authors will be contacted for clarification. If clarification cannot be obtained, studies will be classified by two independent reviewers based on available study information, with disagreements resolved through discussion or consultation with a third reviewer. Where appropriate, sensitivity analyses will be undertaken to assess the impact of including such studies.

### 2.2 Study designs

RCTs will be included. Quasi-experimental, observational studies, reviews, case reports, case series, opinions, and commentaries will be excluded.

### 2.3 Other criteria

Studies conducted across the globe and published in English language will be included.

### 2.4 Literature search

An independent systematic literature search will be performed to identify studies that evaluate the correct timing of treatment initiation. The databases “PubMed, Scopus, EMBASE, and the Cochrane Library” will be searched from inception until 30.12.2025. The search strategy was prepared by one of the reviewers and peer reviewed by another external reviewer by adhering to the PRESS guidelines ^20^ (Extended Data: Table S2). Combination of “Medical Subject Headings (MeSH)” and free-text terms were used to frame the search strategy. Search strategies for the included databases are attached as Extended Data (Table S3).

A consensus meeting was conducted to finalize the search strategy. The records retrieved from the searches will be screened using predefined inclusion and exclusion criteria. Additional methods for identifying relevant studies, including manual screening of reference lists, within the eligible studies from the database search, will be undertaken.

### 2.5 Study evaluation and selection

Studies identified through the systematic search will be imported into the Nested Knowledge platform, ^21^ where deduplication will be performed, followed by screening. The initial screening of the titles and abstracts will be performed by two independent reviewers.

Full-text assessment: Potentially eligible studies will undergo full-text retrieval and will be assessed independently by two reviewers to confirm eligibility for inclusion.

Disagreements: Any discrepancies in screening will be resolved through discussion between the two reviewers, and unresolved conflicts will be resolved by consulting a third reviewer.

The reference list of related systematic reviews identified during the screening process and the reference list of eligible primary studies will also be screened by two reviewers to identify any further eligible studies. Any discrepancies in screening will be resolved through discussion between the two reviewers, and unresolved conflicts will be resolved by consulting a third reviewer.

### 2.6 Data extraction process

Two reviewers independently will extract the data using a process to resolve differences. A data extraction sheet will be constructed in MS Excel and piloted before the data extraction. Two independent reviewers will have a consensus meeting at the end of the independent data extraction to resolve differences. Any conflicts that cannot be resolved will be adjudicated by a third reviewer.

Data extraction will include information on study identifiers, location, study population characteristics (age, sex, co-morbidities, pregnant/non-pregnancy, diagnosis methods, diagnostic criteria used in each study, including TSH thresholds, free thyroxine (FT4) criteria, pregnancy-specific definitions where applicable, and any other relevant diagnostic parameters), intervention group details (demography, dose, frequency, type of drug, duration, and any other relevant information as disclosed in the studies), comparator group details (demography, monitoring details, any other relevant information reported by the studies), outcomes such as progression from subclinical to overt hypothyroidism, prevention of disease-related complications (including mortality, maternal, fetal, neonatal, and other subgroup-specific outcomes), and adverse effects associated with LT4 therapy. Data on improvements in hypothyroidism-related symptoms, changes in health-related quality of life, and health system outcomes, such as cost-effectiveness, feasibility, acceptability, and equity, will also be extracted where reported. Multiple publications from the same study will be identified by comparing trial registration numbers and study characteristics, including authors, study centres/settings, recruitment periods, sample sizes, and intervention and comparator groups. Reports identified as arising from the same study will be linked and considered as a single study. Information from more than one report may be used when they provide data on different outcomes or follow-up periods; however, the same participants will not be included more than once in a meta-analysis. If it is unclear whether publications relate to the same study, the study authors will be contacted for clarification, where feasible.

### 2.7 Risk of bias assessment

The risk of bias for the “randomized controlled trials (RCTs)” will be evaluated through Cochrane’s ROB 2.0, ^22^ independently by at least two reviewers, with a process to resolve differences. Two independent reviewers will have a consensus meeting at the end of the independent risk of bias assessment to resolve the differences. Any conflicts that cannot be resolved will be adjudicated by a third reviewer.

### 2.8 Data analysis

Pooled estimates will be calculated for outcomes where studies are considered sufficiently comparable with respect to participants, interventions, comparators, outcome definitions, and study design. Meta-analyses will be conducted separately for clinically meaningful population groups (e.g., Neonates, children, reproductive status including pregnancy and lactation, women with infertility, the geriatric population, and any other clinically distinct disease/ population group identified during the review). Population groups will be combined only where there is sufficient clinical and methodological similarity and a clear scientific justification for quantitative pooling. Separate meta-analysis will be conducted according to the type of the control group as well (watchful waiting or delayed initiation, or no treatment (C) on progression to overt hypothyroidism). For dichotomous outcomes (e.g., progression to overt hypothyroidism, adverse events, mortality, maternal or neonatal outcomes) assessed at a defined follow-up time, risk ratios (RRs) with 95% confidence intervals (CIs) will be the preferred effect measure. Studies reporting only odds ratios (ORs), where an RR cannot be reliably derived, will be synthesized separately using ORs. For time-to-event outcomes, hazard ratios (HRs) with 95% CIs will be synthesized separately and will not be pooled with RRs or ORs. Clinically comparable follow-up time points will be analyzed separately where appropriate. “Mean difference (MD)” or “standardized mean difference (SMD)” with 95% CI will be calculated for continuous outcomes such as symptom scores and quality-of-life measures. Statistical heterogeneity will be quantified using the I ^2^ statistic. Where no statistical heterogeneity is observed (I ^2^ = 0%) and studies are considered clinically and methodologically comparable, pooled estimates will be calculated using a fixed-effect model. Where residual statistical heterogeneity is present (I ^2^ > 0%) after accounting for relevant clinical and methodological differences, a random-effects model will be used. Sub-group analysis and meta-regression will be performed to explore heterogeneity, if present. ^23^ Where substantial heterogeneity is identified, potential sources will be explored through predefined subgroup analyses (co-morbidities, symptom status, TSH thresholds used for diagnosis (e.g., 4–7 mIU/L, 7–10 mIU/L, >10 mIU/L, or any other cut-off specified by the included studies for the diagnosis of subclinical hypothyroidism), autoimmune status, between different comparators, treatment duration, follow-up duration, environmental or geographic, or any other factor identified during the review) and, where sufficient studies are available (>10 studies per meta-analyzable outcome), meta-regression will be undertaken. Sensitivity analyses will be performed using a leave-one-out approach to examine whether the overall results are unduly influenced by a single study. Publication bias assessment using funnel plots and the Eggers test index will be performed if more than 10 eligible studies are included in the meta-analysis. Statistical evaluations will be performed using RevMan Web. ^24^

Narrative synthesis will be undertaken when quantitative pooling is not appropriate, including where studies are judged to be substantially heterogeneous with respect to participants, interventions, comparators, outcome definitions, study design, or timing of outcome assessment, or where insufficient data are available to calculate pooled effect estimates. In such cases, findings will be synthesised using a structured narrative approach and grouped into clinically meaningful population groups, interventions, and outcomes.

### 2.9 Certainty assessment

“GRADE (Grading of Recommendations, Assessment, Development and Evaluation)” approach will be used for assessing the certainty (confidence) of the evidence for each outcome. The domains considered will include “risk of bias, inconsistency, indirectness, imprecision, and publication bias.” The GRADEpro tool will be used to generate a summary table of certainty in evidence. ^25^

## 3. Discussion

The decision to initiate hormone replacement therapy for SCH has been controversial, with varied evidence profiles in different population groups. For pregnant women, it has been recommended to initiate LT4 therapy following diagnosis in cases of SCH, ^19^ whereas recommendations for adult SCH patients are against early initiation of LT4 therapy. ^17^ However, even within pregnancy outcomes, systematic reviews have reported differential pooled estimates, with favorable (reduced APOs), ^14^ and neutral (no change in APOs), ^26^ findings for LT4 therapy in SCH. The impact of co-morbidities on the effect of early initiation of LT4 in SCH patients and specific outcomes, such as cardiovascular events, has been studied by certain primary studies. ^27, 28^ Studies have also assessed age group specific impact of the LT4 therapy in SCH outcomes, as well. ^28, 29^ Effect of the early LT4 therapy in SCH infertile women on their fertility outcomes has also been reported in the past. ^30^

The lack of certainty assessment (e.g., GRADE profile) in the outcomes reported is a lacuna in the existing systematic reviews on this topic, ^14, 15, 18, 31^ which seriously limits the transferability for guideline development. Through the current systematic review, we intend to provide a comprehensive, updated (until December 2025) evidence profile on the effect of early initiation of LT4 therapy on important and critical patient outcomes (progression to overt hypothyroidism, mortality, complications, quality of life, acceptability, and costs) for multiple population groups (adults, non-pregnant women, infertile women, pregnant women, neonates, children, and co-morbidities). Subgroup analysis was planned in this review to explore the effect modification potential of all critical factors (age, TSH range for SCH diagnosis, symptom status, autoimmunity, and type of control group) on the direction and magnitude of the association between early initiation of LT4 treatment and outcomes in SCH patients. GRADE profiling of critical and important outcomes will be performed in the index systematic review, which can provide certainty in the evidence pooled in the review.

Overall, the findings of the systematic review can inform the framing of the clinical guidelines for SCH management for all population groups, with or without conditional recommendations, as per the variations that may arise due to the subgroup analysis. The review can also reveal the differential effects, if any, according to the severity of the SCH (TSH levels and symptom status), which can enable layered recommendations instead of a blanket recommendation. The outputs from the SRMA can ensure robust guidelines centered on systematically collated, transparent, and critically appraised evidence, which in turn can enhance clinical practice and patient outcomes.

The SRMA also intends to bring out gaps in the existing literature (in terms of lack of adequate evidence in specific populations, specific outcomes, sub-groups, and quality of the studies conducted thus far). This can enable the formulation of research questions for future studies on the management of SCH using early initiation of LT4 to address these gaps.

## Ethical considerations

Not applicable, since it is systematic review of published articles.

## Use of Artificial Intelligence (AI) tools declaration

The authors declare that they have not used AI tools in the creation of this article.

## Data Availability

No data are associated with this article. Figshare: PRESS 2015 Guideline Evidence-Based Checklist for search strategy (Table S2) https://doi.org/10.6084/m9.figshare.31820230.v1. ^32^ Figshare: Search strategies for the databases (Table S3) https://doi.org/10.6084/m9.figshare.31820242.v2. ^33^ Preferred Reporting Items for Systematic Reviews and Meta-Analyses Protocols (PRISMA-P 2015) (Table S1) https://doi.org/10.6084/m9.figshare.31820203.v1. ^34^ Data are available under the terms of the Creative Commons Attribution 4.0 International license (CC-BY 4.0).
