# Supplementary material for: Early Initiation of Thyroid Hormone Therapy Versus Watchful Waiting or Delayed Treatment or no Treatment in Subclinical Hypothyroidism: A Systematic Review and Meta-analysis Protocol
Source: F1000Res. 2026 Sep 29;15:555. Originally published 2026 Apr 18. [Version 3] doi: 10.12688/f1000research.179360.3 (PMC13613001; doi:10.12688/f1000research.179360.3)
Supplement: Peer review history

# Reviewer report: Reviewer response for version 2

**DOI:** 10.5256/f1000research.207851.r520102

**Published:** 2026-09-24

**Authors:** Kanica Kaushal

**Affiliations:** 1 Institute of Liver and Biliary Sciences, New Delhi, New Delhi, India

Reviewer Comments

 Overall, this is a well-structured and clinically important protocol. The PROSPERO registration, PRISMA-P adherence, detailed PICO framework, and provision of extended data (PRESS checklist, search strategies) via Figshare are commendable and support transparency. A few additional methodological points are worth addressing before the review is conducted:

 1.    Section 2.8 allows RR, OR, or HR for dichotomous outcomes but does not specify how these will be handled when different included trials report different metrics for the same outcome. Since RR, OR, and HR are not interchangeable (HR incorporates time-to-event structure), pooling them together without a pre-specified rule risks a methodological error. Please state a preferred effect metric per outcome and how studies reporting a different metric will be handled (conversion method, or a separate analysis stratum).

 2.    The current wording implies REM will be used only when I²>50%, with a fixed-effect model used otherwise. Since I² is imprecise with a small number of studies and the fixed-effect model's single-true-effect assumption is rarely justified even with low observed heterogeneity, most current methodological guidance (e.g., Cochrane Handbook) recommends REM as the default regardless of I². Please reconsider this decision rule or provide justification for the I²-based switch.

 3.    The pre-specified TSH bands (4–7, 7–10, >10 mIU/L) appear intended as a universal subgrouping scheme, but pregnancy-specific TSH reference ranges (trimester-specific, typically ~2.5–4.0 mIU/L upper limit) differ substantially from non-pregnant thresholds. Applying the same bands to pregnant and non-pregnant subgroups could produce clinically meaningless strata for the pregnancy population. Please specify a separate TSH-banding scheme for pregnancy-specific analyses.

 4.    The Discussion states the review will provide an evidence profile including “mortality”, but Table 1’s Critical/Important outcomes list does not include mortality anywhere. Please resolve this inconsistency, either add mortality explicitly to Table 1's outcome list or remove it from the Discussion framing.

 5.    No process is described for identifying multiple publications arising from the same trial (e.g., primary report plus secondary or long-term follow-up analyses), which risks double-counting participants in the meta-analysis. Please add a brief statement on how this will be identified and handled (e.g., cross-checking trial registration numbers, contacting authors when unclear).

Is the study design appropriate for the research question?

Yes

Is the rationale for, and objectives of, the study clearly described?

Yes

Are sufficient details of the methods provided to allow replication by others?

Yes

Are the datasets clearly presented in a useable and accessible format?

Yes

Reviewer Expertise:

Implementation research, evidence based guidelines,

I confirm that I have read this submission and believe that I have an appropriate level of expertise to confirm that it is of an acceptable scientific standard.

# Response

**Published:** 2026-09-24

**Authors:** Aravind Gandhi P

**General reviewer comment: Overall, this is a well-structured and clinically important protocol. The PROSPERO registration, PRISMA-P adherence, detailed PICO framework, and provision of extended data (PRESS checklist, search strategies) via Figshare are commendable and support transparency. A few additional methodological points are worth addressing before the review is conducted:**

**Author response: **Thank you for the positive comments and the methodological issues pointed out. We have addressed these points in the revised manuscript and have added a comment-specific response in this file. These suggestions were useful in refining the methods and making the proposed analysis more transparent.

**Reviewer comment 1: Section 2.8 allows RR, OR, or HR for dichotomous outcomes but does not specify how these will be handled when different included trials report different metrics for the same outcome. Since RR, OR, and HR are not interchangeable (HR incorporates time-to-event structure), pooling them together without a pre-specified rule risks a methodological error. Please state a preferred effect metric per outcome and how studies reporting a different metric will be handled (conversion method, or a separate analysis stratum).**

**Author response: **We thank the reviewer for highlighting this important issue. We agree that RR, OR, and HR are not interchangeable and should not be pooled together as if they represented the same effect measure. We have revised the Data Analysis section to prespecify the approach. For dichotomous outcomes assessed at a defined follow-up time, risk ratio (RR) with 95% confidence interval (CI) will be the preferred effect measure. Where sufficient event and participant data are available, RRs will be calculated from the reported data. Studies for which only an odds ratio (OR) is available and an RR cannot be reliably derived will be analysed separately using ORs rather than pooled directly with RRs. For time-to-event outcomes, hazard ratios (HRs) with 95% CIs will be synthesised separately and will not be combined with RRs or ORs. Where studies report outcomes at substantially different follow-up periods, clinically comparable time points will be synthesised separately. These clarifications have been incorporated into the Data Analysis section.

*Change made in the manuscript (Data analysis section): For dichotomous outcomes assessed at a defined follow-up time, risk ratios (RRs) with 95% confidence intervals (CIs) will be the preferred effect measure. Studies reporting only odds ratios (ORs), where an RR cannot be reliably derived, will be synthesized separately using ORs. For time-to-event outcomes, hazard ratios (HRs) with 95% CIs will be synthesized separately and will not be pooled with RRs or ORs. Clinically comparable follow-up time points will be analyzed separately where appropriate.*

**Reviewer comment 2: The current wording implies REM will be used only when I²>50%, with a fixed-effect model used otherwise. Since I² is imprecise with a small number of studies and the fixed-effect model's single-true-effect assumption is rarely justified even with low observed heterogeneity, most current methodological guidance (e.g., Cochrane Handbook) recommends REM as the default regardless of I². Please reconsider this decision rule or provide justification for the I²-based switch.**

**Author response: **We thank the reviewer for this important methodological observation. We would like to point out that the Cochrane Handbook does not specify that a fixed-effect model cannot be used when only a small number of studies are available. However, we agree with the reviewer that there is no definitive binary I² cut-off for choosing between fixed-effect and random-effects models and that model selection should not be based solely on an arbitrary I² threshold. Accordingly, we have mentioned our analytical approach. In the present systematic review and meta-analysis, studies will first be assessed for clinical and methodological comparability. Population groups (e.g., adults, pregnant women, older adults), comparator groups (watchful waiting, delayed initiation, or no treatment), and outcomes will be pooled only when they are sufficiently similar to justify quantitative synthesis. After accounting for these characteristics, if residual statistical heterogeneity is present (I² >0%), a random-effects model will be used for pooling. Where no statistical heterogeneity is observed (I² = 0%), and the included studies are considered clinically and methodologically comparable, a fixed-effect model will be used. The previous I² >50% criterion for selecting the random-effects model has therefore been removed from the Data Analysis section.

*Change made in the manuscript (Data analysis section): Meta-analyses will be conducted separately for clinically meaningful population groups (e.g., Neonates, children, reproductive status including pregnancy and lactation, women with infertility, the geriatric population, and any other clinically distinct disease/ population group identified during the review). Population groups will be combined only where there is sufficient clinical and methodological similarity and a clear scientific justification for quantitative pooling. Separate meta-analysis will be conducted according to the type of the control group as well (watchful waiting or delayed initiation, or no treatment (C) on progression to overt hypothyroidism). Statistical heterogeneity will be quantified using the I² statistic. Where no statistical heterogeneity is observed (I² = 0%) and studies are considered clinically and methodologically comparable, pooled estimates will be calculated using a fixed-effect model. Where residual statistical heterogeneity is present (I²> 0%) after accounting for relevant clinical and methodological differences, a random-effects model will be used. Where substantial heterogeneity is identified, potential sources will be explored through predefined subgroup analyses (co-morbidities, symptom status, TSH thresholds used for diagnosis (e.g., 4–7 mIU/L, 7–10 mIU/L, >10 mIU/L, or any other cut-off specified by the included studies for the diagnosis of subclinical hypothyroidism) , autoimmune status, between different comparators, treatment duration, follow-up duration, environmental or geographic, or any other factor identified during the review) and, where sufficient studies are available (>10 studies per meta-analyzable outcome), meta-regression will be undertaken.*

**Reviewer comment 3: The pre-specified TSH bands (4–7, 7–10, >10 mIU/L) appear intended as a universal subgrouping scheme, but pregnancy-specific TSH reference ranges (trimester-specific, typically ~2.5–4.0 mIU/L upper limit) differ substantially from non-pregnant thresholds. Applying the same bands to pregnant and non-pregnant subgroups could produce clinically meaningless strata for the pregnancy population. Please specify a separate TSH-banding scheme for pregnancy-specific analyses.**

**Author response: **We thank the reviewer for this important observation. The initially specified TSH categories (4–7, 7–10, and >10 mIU/L) were based on the author team's clinical experience and preliminary review of the literature and were intended as potential categories for subgroup analysis rather than as universal diagnostic thresholds for subclinical hypothyroidism. We agree with the reviewer that the TSH thresholds used to diagnose subclinical hypothyroidism can vary according to the population, clinical setting, pregnancy/reproductive status, geographical setting, and diagnostic criteria or guidelines followed by individual studies. Accordingly, we have revised the protocol to avoid restricting subgroup analyses to these prespecified TSH categories. In addition to the proposed categories, we have included an open criterion of “any other TSH cut-off specified by the included studies for the diagnosis of subclinical hypothyroidism.” The exact diagnostic threshold and criteria used in each study will be extracted during data extraction. For population groups with specific diagnostic thresholds, particularly pregnant women, subgrouping will be based on the population- or pregnancy-specific diagnostic criteria and TSH thresholds reported by the included studies rather than applying the proposed general TSH categories uniformly. These changes have been incorporated into the Data Extraction and Data Analysis sections.

*Change made in the manuscript (Data Analysis): Where substantial heterogeneity is identified, potential sources will be explored through predefined subgroup analyses (co-morbidities, symptom status, TSH thresholds used for diagnosis (e.g., 4–7 mIU/L, 7–10 mIU/L, >10 mIU/L, or any other cut-off specified by the included studies for the diagnosis of subclinical hypothyroidism) , autoimmune status, between different comparators, treatment duration, follow-up duration, environmental or geographic, or any other factor identified during the review) and, where sufficient studies are available (>10 studies per meta-analyzable outcome), meta-regression will be undertaken.*

**Reviewer comment 4: The Discussion states the review will provide an evidence profile including “mortality”, but Table 1’s Critical/Important outcomes list does not include mortality anywhere. Please resolve this inconsistency, either add mortality explicitly to Table 1's outcome list or remove it from the Discussion framing.**

**Author response: **We thank the reviewer for identifying this inconsistency. Mortality is a clinically relevant outcome for this review, particularly for long-term follow-up and specific population groups. Accordingly, we added mortality to the outcome list in Table 1 and ensured it is consistently included, where relevant in the manuscript.

*Change made in the manuscript (Table 1 – Outcomes): “Mortality (all-cause and, where reported, cause-specific mortality)” has been added among the prespecified outcomes.*

**Reviewer comment 5: No process is described for identifying multiple publications arising from the same trial (e.g., primary report plus secondary or long-term follow-up analyses), which risks double-counting participants in the meta-analysis. Please add a brief statement on how this will be identified and handled (e.g., cross-checking trial registration numbers, contacting authors when unclear).**

**Author response:** Thank you for pointing this out. We have now added a process for identifying multiple publications from the same study. We will compare trial registration numbers and study details such as the authors, study centers, recruitment period, sample size, and intervention and comparator groups to identify reports that may have originated from the same study. Such publications will be linked and considered as a single study so that the same participants are not included more than once in a meta-analysis. If different reports provide information on different outcomes or follow-up periods, the relevant information will be taken from each report. Where it is not clear whether two publications are from the same study, we will contact the study authors for clarification, where feasible. This has now been added to the Data Extraction Process section.

*Change made in the manuscript (Data Extraction Process): Multiple publications from the same study will be identified by comparing trial registration numbers and study characteristics, including authors, study centres/settings, recruitment periods, sample sizes, and intervention and comparator groups. Reports identified as arising from the same study will be linked and considered as a single study. Information from more than one report may be used when they provide data on different outcomes or follow-up periods; however, the same participants will not be included more than once in a meta-analysis. If it is unclear whether publications relate to the same study, the study authors will be contacted for clarification, where feasible.*

# Reviewer report: Reviewer response for version 2

**DOI:** 10.5256/f1000research.207851.r510771

**Published:** 2026-08-21

**Authors:** Haider Ayad Alidrisi

**Affiliations:** 1 University of Basrah College of Medicine, Basrah, Basra Governorate, Iraq; 2 Faiha Specialized Diabetes, Endocrine, and Metabolism Center, Iraq, Iraq

Thanks for the author for their valuable revisions and clarifications.

Is the study design appropriate for the research question?

Yes

Is the rationale for, and objectives of, the study clearly described?

Yes

Are sufficient details of the methods provided to allow replication by others?

Partly

Are the datasets clearly presented in a useable and accessible format?

Not applicable

Reviewer Expertise:

Endocrine disorders

I confirm that I have read this submission and believe that I have an appropriate level of expertise to confirm that it is of an acceptable scientific standard.

# Reviewer report: Reviewer response for version 1

**DOI:** 10.5256/f1000research.197865.r488634

**Published:** 2026-07-30

**Authors:** Haider Ayad Alidrisi

**Affiliations:** 1 University of Basrah College of Medicine, Basrah, Basra Governorate, Iraq; 2 Faiha Specialized Diabetes, Endocrine, and Metabolism Center, Iraq, Iraq

This article presents a protocol for a systematic review and meta-analysis evaluating early initiation of thyroid hormone therapy, specifically levothyroxine, compared with watchful waiting, delayed treatment, or no treatment in individuals with subclinical hypothyroidism. The proposed review intends to assess whether early treatment reduces progression to overt hypothyroidism, prevents complications, improves symptoms and quality of life, and affects adverse outcomes across several population groups, including adults, older adults, pregnant women, infertile women, neonates, children, postpartum women, and individuals with comorbidities.

 The protocol is registered in PROSPERO and follows PRISMA-P guidance. The authors plan to include randomized controlled trials, search PubMed, Scopus, EMBASE, and the Cochrane Library from inception to 30 December 2025, screen studies independently in duplicate, assess risk of bias using Cochrane RoB 2.0, conduct meta-analysis where appropriate, explore heterogeneity through subgroup analyses and meta-regression, and assess certainty of evidence using GRADE.

 Overall, this is a clinically relevant and potentially useful protocol addressing an important area of uncertainty in endocrine and reproductive medicine. The manuscript is generally well structured and the rationale for the review is clear. However, several methodological details require clarification before the protocol can be considered fully reproducible and scientifically robust.

 Although the rationale and objectives are clear, the authors could strengthen this section by ensuring consistent wording throughout the manuscript. At times, the review is described as assessing “early initiation of treatment,” while elsewhere it is described more broadly as assessing “LT4 therapy.” The authors should clarify whether the key research question is specifically about timing of treatment initiation or about LT4 treatment versus non-treatment more generally.

 The authors should specify that meta-analyses will be conducted separately by clinically meaningful population groups unless there is a clear justification for combining them. They should also define in advance when narrative synthesis will be preferred over quantitative pooling.

 The authors should provide an operational definition of early initiation, such as treatment started at diagnosis or within a specified time window after diagnosis. They should also describe how they will classify trials that do not clearly report timing of initiation.

 The authors should specify how they will handle different diagnostic thresholds. For example, they could accept each trial’s definition but extract diagnostic criteria in detail and perform subgroup or sensitivity analyses by TSH threshold, pregnancy-specific criteria, and population group.

 As this is a systematic review protocol, it is reasonable that no underlying study dataset is yet associated with the article. The authors provide extended data through Figshare, including the PRISMA-P checklist, PRESS checklist, and search strategies, with DOIs and a CC-BY 4.0 licence. This supports accessibility and reuse.

 Points to consider

 · Define “early initiation” of LT4 therapy operationally.

 · Clarify diagnostic criteria for SCH across different populations.

 · Define comparator groups clearly and state whether they will be analyzed separately or combined.

 · Specify how clinically heterogeneous populations will be analyzed, preferably with population-specific syntheses.

 · Prioritize subgroup analyses and specify feasibility criteria for meta-regression.

Is the study design appropriate for the research question?

Yes

Is the rationale for, and objectives of, the study clearly described?

Yes

Are sufficient details of the methods provided to allow replication by others?

Partly

Are the datasets clearly presented in a useable and accessible format?

Not applicable

Reviewer Expertise:

Endocrine disorders

I confirm that I have read this submission and believe that I have an appropriate level of expertise to confirm that it is of an acceptable scientific standard, however I have significant reservations, as outlined above.

# Response

**Published:** 2026-08-01

**Authors:** Aravind Gandhi P

**General reviewer comment**: Overall, this is a clinically relevant and potentially useful protocol addressing an important area of uncertainty in endocrine and reproductive medicine. The manuscript is generally well structured and the rationale for the review is clear. However, several methodological details require clarification before the protocol can be considered fully reproducible and scientifically robust.

**Author response**: We sincerely thank the reviewer for the suggestions. We have revised the manuscript in response to the comments, and this has helped us improve the scientific rigor of the protocol.

**Reviewer comment 1**: Although the rationale and objectives are clear, the authors could strengthen this section by ensuring consistent wording throughout the manuscript. At times, the review is described as assessing “early initiation of treatment,” while elsewhere it is described more broadly as assessing “LT4 therapy.” The authors should clarify whether the key research question is specifically about timing of treatment initiation or about LT4 treatment versus non-treatment more generally.

**Author response 1**: Thank you for the observation. The primary research question of this review is to evaluate the timing of treatment initiation, i.e., effects of early initiation of thyroid hormone therapy compared with watchful waiting, delayed initiation of therapy, or no treatment in individuals with subclinical hypothyroidism. Accordingly, we have ensured uniformity in conveying this in the revised manuscript. Watchful waiting, or delayed initiation, or no treatment has been taken as the comparator/control group. The mention of ‘LT4 therapy’ in general in SCH is limited to the beginning of the introduction section, where we provide the background/rationality for hormone replacement therapy in SCH and discuss existing studies in specific groups on the treatment of SCH (pregnant women). However, in the later sections of the manuscript, we have currently revised it to reflect the specific intervention of interest, ‘early initiation of the LT4’. ‘Early initiation of treatment’ refers to ‘Early initiation of thyroid hormone therapy’ only. The same has been clarified in the research question as well. (Introduction, Research Question and Discussion)

**Reviewer comment 2**: The authors should specify that meta-analyses will be conducted separately by clinically meaningful population groups unless there is a clear justification for combining them. They should also define in advance when narrative synthesis will be preferred over quantitative pooling.

**Author response 2**: Thank you for the observation. The Data Analysis section has been revised to clarify that quantitative syntheses will be conducted separately for clinically meaningful population groups (e.g., based on age, life stage/reproductive status, pregnancy status, and other clinically relevant characteristics). Population groups will be combined only where there are sufficient clinical and methodological similarity and a clear scientific justification for quantitative pooling. We have also prespecified the situations in which narrative synthesis will be undertaken, including when studies are too heterogeneous with respect to participants, interventions, comparators, outcome definitions, or study design, or when insufficient data are available for quantitative pooling. (Data analysis)

**Reviewer comment 3**: The authors should provide an operational definition of early initiation, such as treatment started at diagnosis or within a specified time window after diagnosis. They should also describe how they will classify trials that do not clearly report timing of initiation.

**Author response 3**: Thank you for this valuable suggestion. The protocol has been revised to provide an operational definition of early initiation. For the purpose of this review, early initiation will refer to thyroid hormone therapy initiated immediately following the diagnosis of subclinical hypothyroidism, without a planned period of observation or delayed treatment. Studies comparing this strategy with watchful waiting, delayed initiation, or no treatment will be eligible. We have also clarified that when the timing of treatment initiation is not explicitly reported, the study will be assessed based on the intervention description and treatment protocol. If the timing remains unclear after reviewing the full text and supplementary materials, the study authors will be contacted for clarification. If clarification cannot be obtained, the study will be classified according to the best available evidence by two independent reviewers, with disagreements resolved by consensus or a third reviewer, and the potential impact of such studies will be explored through sensitivity analyses where appropriate. The details have been included in the revised manuscript. (Research question)

**Reviewer comment 4**: The authors should specify how they will handle different diagnostic thresholds. For example, they could accept each trial’s definition but extract diagnostic criteria in detail and perform subgroup or sensitivity analyses by TSH threshold, pregnancy-specific criteria, and population group.

**Author response 4**: Thank you for this valuable suggestion. Given the expected variability in the diagnostic criteria for subclinical hypothyroidism across different populations, geographical settings, time periods, and evolving clinical guidelines, we will include all eligible studies irrespective of the diagnostic thresholds used, provided the study authors classify participants as having subclinical hypothyroidism. The diagnostic criteria used in each study, including TSH thresholds, free thyroxine (FT4) criteria, pregnancy-specific definitions (where applicable), and any other relevant diagnostic parameters, will be extracted in detail during data extraction. To explore the potential influence of differing diagnostic definitions, predefined subgroup analyses will be undertaken based on TSH thresholds used for diagnosis (e.g., 4–7 mIU/L, 7–10 mIU/L, >10 mIU/L), pregnancy-specific versus non-pregnant populations, and other clinically relevant population groups, where sufficient data are available. Where appropriate, sensitivity analyses will also be performed to assess the robustness of the findings to differences in diagnostic criteria.

 We have added these points under the methods section of the revised manuscript. (Research Question, Data Extraction, and Data Analysis)

**Reviewer comment 5**: As this is a systematic review protocol, it is reasonable that no underlying study dataset is yet associated with the article. The authors provide extended data through Figshare, including the PRISMA-P checklist, PRESS checklist, and search strategies, with DOIs and a CC-BY 4.0 licence. This supports accessibility and reuse.

**Author response 5**: Thank you for the observations and positive comments.

**Reviewer comment 6**: Points to consider

 · Define “early initiation” of LT4 therapy operationally.

 · Clarify diagnostic criteria for SCH across different populations.

 · Define comparator groups clearly and state whether they will be analyzed separately or combined.

 · Specify how clinically heterogeneous populations will be analyzed, preferably with population-specific syntheses.

 · Prioritize subgroup analyses and specify feasibility criteria for meta-regression.

**Author response 6**: We thank the reviewer for these helpful summary recommendations. The manuscript has been revised accordingly. Specifically, we have:

- Operationally defined "early initiation" of thyroid hormone therapy and clarified the eligibility of studies with unclear timing of treatment initiation (Research Question).
- Clarified the approach to varying diagnostic criteria for subclinical hypothyroidism across different populations by accepting the study authors' definitions, extracting detailed diagnostic criteria, and planning subgroup and sensitivity analyses based on diagnostic thresholds and population groups (Research Question, Data Extraction, and Data Analysis).
- Defined the comparator groups (watchful waiting, delayed initiation of therapy, and no treatment) and specified that they will be analysed separately where appropriate (Data Analysis).
- We have specified that clinically heterogeneous populations will be synthesised separately by clinically meaningful population groups, with quantitative pooling undertaken only where there is sufficient clinical and methodological similarity (Data Analysis).
- Refined the statistical analysis plan by prespecifying subgroup analyses and defining the feasibility criterion for meta-regression (i.e., where sufficient studies are available) (Data Analysis).
